# Analgesic effects of the COX-2 inhibitor parecoxib on surgical pain through suppression of spinal ERK signaling

**DOI:** 10.3892/etm.2013.1118

**Published:** 2013-05-15

**Authors:** YA-JING GUO, XU-DAN SHI, DI FU, YONG YANG, YA-PING WANG, RU-PING DAI

**Affiliations:** 1 Institute of Combined Traditional Chinese and Western Medicine, Xiangya Hospital of Central South University, Changsha, Hunan 410011;; 2 Department of Anesthesia, Xiangya Hospital of Central South University, Changsha, Hunan 410011;; 3 Department of Anesthesia, The Second Xiangya Hospital of Central South University, Changsha, Hunan 410078, P.R. China

**Keywords:** postoperative pain, extracellular signal-regulated kinase, spinal cord, parecoxib, cyclooxygenase

## Abstract

Cyclooxygenase (COX)-2 inhibitors are widely used for postoperative pain control in clinical practice. However, it is unknown whether spinal sensitization is involved in the analgesic effects of COX-2 inhibitors on surgical pain. Extracellular signal-regulated kinase (ERK) in the spinal cord is implicated in various types of pain, including surgical pain. The present study investigated the role of spinal ERK signaling in the analgesic effect of the COX-2 inhibitor parecoxib on surgical pain. Surgical pain was produced in rats by surgical incision of the hind paw. Phosphorylated (p)-ERK1/2 expression was determined by immunohistochemistry. Pain hypersensitivity was evaluated by measuring the paw withdrawal threshold using the von Frey test. The selective COX-2 inhibitor parecoxib was delivered 20 min before or 20 min after the incision by intraperitoneal injection. Pretreatment with parecoxib markedly attenuated the pain hypersensitivity induced by incision. However, post-treatment with parecoxib produced minimal analgesic effects. Parecoxib inhibited the increase in spinal p-ERK expression following surgical incision. The present study thus suggests that the COX-2 inhibitor parecoxib exerts its analgesic effect on surgical pain through the inhibition of neuronal ERK activation in the spinal cord. COX-2 inhibitor delivery prior to surgery has more potent analgesic effects, suggesting the advantage of preventive analgesia for post-operative pain control.

## Introduction

Clinially, pain resulting from surgical trauma (postoperative pain) is a critical challenge for perioperative management (1,2). Current pharmacological treatments of postoperative pain include the use of opioids, non-steroidal anti-inflammatory drugs (NSAIDs) and other drugs, including tramadol and ketamine. NSAIDs exert their analgesic effects through the inhibition of cyclooxygenase (COX), a rate-limiting enzyme that catalyzes the conversion of arachidonic acid to prostaglandins (PGs). COX is composed of two isoforms, COX-1 and COX-2, which are constitutively expressed in the spinal cord. In clinial practice, COX-2 inhibitors are widely used for postoperative pain control, since they have a similar analgesic effect to NSAIDs without the gastrointestinal side-effects and antiplatelet effects (3,4). Systemic delivery of the COX-2 inhibitor parecoxib attenuates the pain score and reduces the consumption of morphine in patients undergoing surgery (5). It is considered that COX-2 inhibitors produce analgesic effects by blocking peripheral sensitization through the inhibition of the production of COX and prostaglandin E2 (PGE2) in the local inflammatory tissue. However, it is unknown whether central sensitization, in particular spinal sensitization, is also involved in the analgesic effect of COX-2 inhibitors.

In inflammatory pain, spinal sensitization plays an important role in the analgesic effect of COX-2. In complete Freund’s adjuvant-induced inflammatory pain, COX-2 is significantly upregulated in the spinal cord (6). In addition, intrathecal delivery of selective COX-2, but not COX-1 inhibitors dramatically reduces the mechanical allodynia and thermal hyperalgesia in various types of inflammatory pain (6,7). In contrast to inflammatory pain, COX-2 expression in the spinal cord is only mildly upregulated in response to surgical incision. Intrathecal delivery of a COX-2 inhibitor has only minimal effects on postoperative pain hypersensitivity (8). These experimental studies suggest that spinal COX-2 may not play an important role in surgical pain. However, a clinical study demonstrated that COX-2 inhibitor administration reduces the visual analog scale pain score and the consumption of opioid drugs in patients postoperatively (9). The analgesic effect of COX-2 in postoperative pain may be associated with the reduction of PGE2 levels in the cerebrospinal fluid (CSF) or local tissue (10). The results of the experimental and clinical studies strongly suggest that the systemic delivery of COX-2 inhibitors produces an analgesic effect through an indirect spinal mechanism.

Extracellular signal-regulated kinase (ERK) in the spinal cord has been implicated in pain processing. In neuropathic and inflammatory pain, activation of ERK in the spinal cord was observed and inhibiting the activation of ERK markedly reduced the pain behavior (11,12). Our previous study demonstrated that phosphorylated (p)-ERK in the spinal cord is also transiently activated following hind paw incision (13). The activation of p-ERK reached a peak level at 5 min after incision and returned to the baseline at 10 min post-incision. Brushing the incised skin at a later time (>10 min after incision) re-activated the expression of p-ERK. Intrathecal delivery of an ERK inhibitor prior to incision, but not post-incision, greatly attenuated pain hypersensitivity in response to the incision (13). These findings suggest that spinal ERK signaling contributes to surgical pain.

The present study thus investigated whether spinal ERK signaling is involved in the analgesic effect of parecoxib, a selective COX-2 inhibitor, on surgical pain. The present study aimed to elucidate the mechanism of the analgesic effect of COX-2 inhibitors on postoperative pain.

## Materials and methods

### Animals

Adult male Sprague-Dawley rats (150–250 g) obtained from Central South University Animal Services (Changsha, China) were used in the present study. All rats were maintained in an air-conditioned (23–26°C, 60–70% relative humidity) vivarium with a 12 h dark/light cycle (light from 8:00 a.m. to 8:00 p.m.). The experimental protocol complied with the National Institutes of Health Guide for the Care and Use of Laboratory Animals and was approved by the Animal Care and Use Committee of Central South University. All efforts were undertaken to minimize the suffering of the rats.

### Surgical preparation and groups

The surgical pain model was established by hind paw incision in the rats. A detailed description of this model in rats has been described in a previous study (14). Briefly, under anesthesia with 1.5% sevoflurane, a 1-cm longitudinal incision was made into the planta skin and deepened to the plantaris muscle. The muscle was then elevated and incised longitudinally (0.5 cm). Then, the skin was closed with 4-0 nylon sutures. A topical triple antibiotic ointment was applied to the hind paw following surgery. Sham surgery was performed under the same procedure with the exception of the incision.

The rats were randomly divided into three groups: parecoxib pretreatment group, parecoxib post-treatment group and saline group (control group). For the parecoxib groups, parecoxib (6 mg/kg; Pharmacia and Upjohn Co., Boston, MA, USA) was intraperitoneally (i.p) injected 20 min before incision (parecoxib pretreatment group) or 20 min after incision (parecoxib post-treatment group), respectively. For the control group, 0.9% saline was injected i.p. For behavior experiments, nocifensive testing was performed prior to incision and 5 min, 10 min, 1 h, 6 h, 1 day and 3 days after incision in the different groups of rats (n=10 for each group with parecoxib pretreatment or post-treatment, n=8 for the saline control group). For immunohistochemical experiments, rats in the control or parecoxib pretreatment groups were sacrificed 5 min after incision. In an independent experiment, rats in the control or parecoxib pretreatment groups 10 min after incision were subjected to brushing of the incised skin followed by immunohistochemical studies.

### Immunohistochemistry

Rats were deeply anesthetized with chloral hydrate (80 mg/kg) and perfused transcardially with 100 ml phosphate-buffered saline (PBS), followed by 4% paraformaldehyde in 0.1 M phosphate buffer. L4–L5 spinal cord segments were fixed for 4 h with 4% paraformaldehyde and then immersed in 20% sucrose in phosphate buffer (pH 7.4) overnight. Transverse spinal cord sections (30 *μ*m) were cut and processed for immunohistochemistry using the ABC method. In brief, sections were mounted on (3-aminopropyl)triethoxysilane-coated slides and incubated with mouse anti-p-ERK antibody (dilution 1:1000; Cell Signaling Technology, Danvers, MA, USA) at room temperature overnight. The secondary reagents used for localization were biotinylated goat anti-mouse IgG and an ABC kit (Vector Laboratories Inc., Burlingame, CA, USA). Diaminobenzidine (DAB) tetrahydrochloride (Sigma, St. Louis, MO, USA) was used as a peroxidase substrate.

### Nociceptive testing

Mechanical allodynia was assayed by measuring the paw withdrawal threshold (PWT) using nylon von Frey filaments (15). In brief, rats were placed on wire mesh platforms in clear cylindrical plastic enclosures. Then, von Frey filaments (0.4–15.1 g) were applied to the wound edge of the incised hind paw or the center of the plantar surface of the unincised paw. According to the up-down method, the test was consecutive (15). In the absence of a paw withdrawal response, a stronger stimulus was applied; otherwise a weaker stimulus was used. Testing proceeded in this manner until four fibers had been applied after the first one that caused a withdrawal response, allowing an estimation of the PWT.

### Quantification and statistical analysis

Eight non-adjacent sections from each specimen of L4–L5 lumbar spinal cord were randomly selected and the expression of p-ERK was determined by counting the positive cells on the L4–L5 spinal superficial dorsal horn (lamina I and II). The investigator during data collection was blind to the treatment that the animals had received. SPSS 13.0 (SPSS, Inc., Chicago, IL, USA) and Prism 5.0 (Graphpad Software Inc., San Diego, CA, USA) were used for statistical analysis. Data are presented as the mean ± standard error of the mean (SEM). Differences between groups were compared with one-way analysis of variance (ANOVA) followed by Dunnett’s post hoc test or Tukey’s post hoc multiple comparison test, where appropriate. P<0.05 was considered to indicate a statistically significant difference.

## Results

### Parecoxib pretreatment attenuates incision-evoked pain hypersensitivity

**Figure 1.**
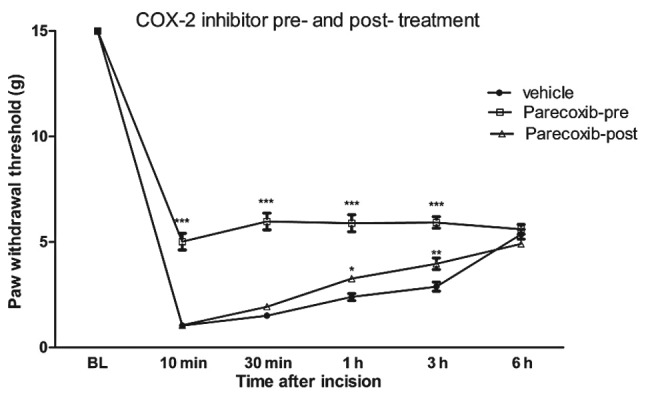
Pre- and post-treatment with the cyclooxygenase (COX)-2 inhibitor parecoxib attenuated the pain behavioral response to hind-paw incision. Parecoxib (6 mg/kg) or vehicle (0.9% saline) was intraperitoneally infused 20 min before (pretreatment) or after (post-treatment) incision, and the behavioral responses to surgical incision were measured by the von Frey test. BL, baseline. ^*^P<0.05, ^**^P<0.01, parecoxib post-treatment group vs. vehicle group; ^***^P<0.001, parecoxib pretreatment group vs. the vehicle group and parecoxib post-treatment group. Data were analyzed by two-way analysis of variance (ANOVA) followed by Tukey’s post hoc test.

Previous studies have clearly shown that hind-paw incision markedly reduces the PWT for >3 days (14,16). The PWT is significantly reduced >6 h after surgical incision as shown in Fig. 1. I.p injection of 6 mg/kg parecoxib 30 min prior to surgery significantly inhibits the reduction in the PWT in response to incision. The analgesic effect of parecoxib is maintained for 3 h. At 6 h after incision, the PWT in the parecoxib pretreatment group was similar to those in the control and parecoxib post-treatment groups. However, parecoxib, when delivered 20 min after incision, has only a minimal attenuating effect on mechanical hypersensitivity following incision. These findings suggest that pretreatment, but not post-treatment with parecoxib attenuates pain hypersensitivity induced by incision.

### Effect of parecoxib pretreatment on p-ERK expression following surgical incision

**Figure 2.**
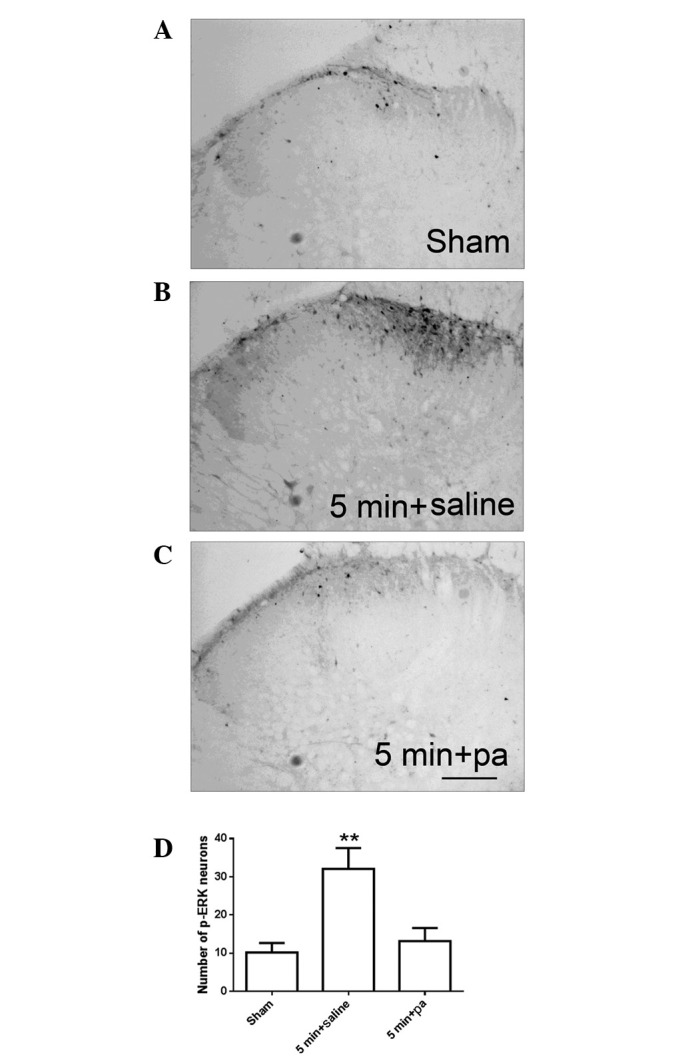
Effect of the cyclooxygenase (COX)-2 inhibitor parecoxib (pa) or saline (control) on phosphorylated extracellular signal-regulated kinase (p-ERK) following surgical incision. (A) Weak expression of p-ERK in the spinal superficial dorsal horns; (B) increased expression of p-ERK at 5 min after incision (5 min + saline); (C) intraperitoneal injection of parecoxib (6 mg/kg) 20 min before hind-paw incision significantly inhibits the activation of p-ERK in the dorsal horns (5 min + pa); (D) quantitative analysis of p-ERK expression by counting the number of p-ERK-immunoreactive neurons. Scale bar, 200 *μ*m. ^**^P<0.01, 5 min + saline group vs. 5 min + pa group or sham group.

Our previous study demonstrated that p-ERK expression is increased following hind paw incision. The increased p-ERK expression was observed 1 min after incision, reaching a peak level 5 min after incision and returning to the baseline 10 min after incision and thereafter (13). As shown in Fig. 2, at 5 min after incision, increased p-ERK immunoreactivity (Fig. 2B) was detected as compared with the sham surgery group (Fig. 2A). Pretreatment with parecoxib significantly inhibited the increase in the number of p-ERK neurons exhibited at 5 min after incision (Fig. 2C and D).

**Figure 3.**
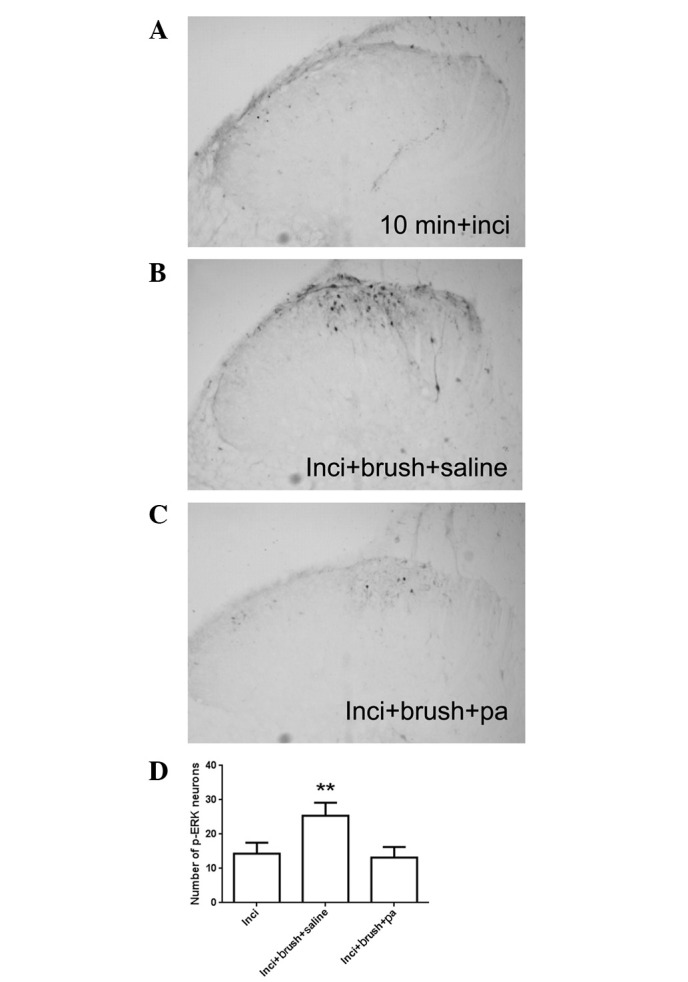
Suppression of brushing-evoked spinal phosphorylated extracellular signal-regulated kinase (p-ERK) activation by parecoxib (pa) pretreatment. (A) Weak expression of p-ERK in the spinal cord at 10 min after incision (10 min + saline); (B) increased expression of spinal p-ERK following brushing (Inci + brush + saline); (C) suppression of the activated spinal p-ERK by parecoxib pretreatment (Inci + brush + pa); (D) quantitative analysis of p-ERK-positive neurons in the different groups. Scale bar, 200 *μ*m. ^**^P<0.01, Inci + brush + saline group vs. Inci + brush + pa group or Inci group. Data were analyzed by one-way analysis of variance (ANOVA) followed by Dunnett’s post hoc test.

Our previous study also identified that at 10 min after incision, when the increased p-ERK expression had returned to baseline, brushing the incised skin re-activated p-ERK expression (13). Confirming our previous results, at 10 min after incision, the p-ERK expression was minimal (Fig. 3A). However, intense staining of p-ERK immunoreactivity was observed in the saline group subjected to brushing at 10 min after incision (Fig. 3B). The re-activated p-ERK expression in the spinal cord is response to brushing was also significantly inhibited by parecoxib pretreatment (Fig. 3C and D). These findings suggest that COX-2 regulates incisional pain through ERK signaling in the spinal cord, at least partially.

## Discussion

A number of studies have shown that systemic administration of a COX-2 inhibitor attenuates postoperative pain and is widely used for pain control following surgery. Experimental and clinical studies have shown that COX-2 inhibitors inhibit the production of PG in the local tissue. The latter in turn elicits sensitization of peripheral nociceptor terminals and pain hypersensitivity (10,17,18). Despite the fact that peripheral sensitization plays important roles in the analgesic effect of COX-2 inhibitors, whether spinal sensitization is involved in the analgesic mechanism of COX-2 inhibitors remains to be determined. Although clinical studies have shown that COX-2 inhibitors also reduce the level of PGE2 in the cerebrospinal fluid (CSF) of patients undergoing vascular surgery, the reduced PGE2 level in the CSF may be the global effect of the reduced production of local PGE2 (10,17).

In the present study, pretreatment with the COX-2 inhibitor parecoxib was demonstrated to significantly inhibit the activation of spinal ERK and attenuate mechanical hypersensitivity following hind paw incision. These findings suggest that the COX-2 inhibitor may exert its analgesic effect through the inhibition of spinal ERK1/2 signaling. Supporting this hypothesis, parecoxib also suppresses the re-activation of spinal ERK in response to brushing following incision. It is well known that ERK in the spinal cord dorsal horn neurons are involved in the induction and maintenance of neural plasticity, including peripheral sensitization and central sensitization (19,20). Only Aδ- or C-fiber stimulation or noxious peripheral stimuli (thermal or mechanical) activate ERK in the dorsal horn, which encodes stimulus intensity (21). The inhibition of spinal ERK by parecoxib suggests that spinal sensitization may also play an important role in the analgesic effects of COX-2 inhibitors (22). Intrathecal delivery of a COX-2 inhibitor has only marginal analgesic effects (8). The present study indicates that the inhibition of spinal ERK by systemic administration of a COX-2 inhibitor may be due to the local effect of the COX-2 inhibitor. In this scenario, the COX-2 inhibitor reduces the production of PGE2 at local inflammatory sites, which in turn reduces the sensitization of the nociceptive nerve fibers. Therefore, the noxious stimuli projecting to the spinal cord superficial dorsal horn neurons is also reduced. These findings together indicate that COX-2 inhibitors exert their analgesic effects through indirect inhibition of spinal sensitization.

In the present study, pretreatment, but not post-treatment with parecoxib produced potent analgesic effects. This finding suggests that pretreatment with a COX-2 inhibitor may provide improved postoperative pain relief, supporting the idea of preventive analgesia clinically. Extensive studies have shown that delivery of analgesia prior to surgical trauma provides improved postoperative pain control (23–25). It is considered that analgesia delivered prior to injury prevents the immediate and long-term effects of noxious operative afferent input, which may induce peripheral and central sensitization and then promote the development of postoperative pain (23–25). However, once peripheral or central sensitization occurs as a response to injury, it may not be totally reversed by analgesia. In the present study, post-treatment of parecoxib had no effect on the transient activation of p-ERK in the spinal cord dorsal horns. The transient activation of p-ERK may subsequently activate multiple downstream pain mediators, which the COX-2 inhibitor may not be able to inhibit. Thus, the findings in the present study strongly support the theory that preventive analgesia is an ideal approach for postoperative pain control.

In conclusion, the present study demonstrated that pretreatment, but not post-treatment with a COX-2 inhibitor significantly attenuates incision-evoked pain hypersensitivity. The COX-2 inhibitor parecoxib suppresses the transient activation of spinal ERK and the reactivation of ERK in response to brushing post-incision, suggesting that COX-2 may regulate incisional pain through spinal ERK signaling.
